# Clinical Phenotype of Cerebral Palsy Depends on the Cause: Is It Really Cerebral Palsy? A Retrospective Study

**DOI:** 10.1177/08830738211059686

**Published:** 2021-12-13

**Authors:** Charlotte Metz, Monika Jaster, Elisabeth Walch, Akosua Sarpong-Bengelsdorf, Angela M. Kaindl, Joanna Schneider

**Affiliations:** 1 14903 Charité-Universitätsmedizin Berlin, Berlin, Germany; 2 14903 Charité-Universitätsmedizin Berlin, Berlin, Germany; 3 522475 Berlin Institute of Health, Berlin, Germany; 4 14903 Charité-Universitätsmedizin Berlin, Institute of Cell and Neurobiology, Berlin, Germany

**Keywords:** cerebral palsy, clinical phenotype, disease cause

## Abstract

Cerebral palsy is the most common motor disability in childhood. Still, the precise definition in terms of causes and timing of the brain damage remains controversial. Several studies examine the clinical phenotype of cerebral palsy types. The aim of our study was to determine to what extent the clinical phenotype of cerebral palsy patients depends on the underlying cause. We retrospectively evaluated the clinical phenotype, abnormalities during pregnancy, and cerebral palsy cause of 384 patients, treated at Charité-Medicine University, between 2015 and 2017. The cause of cerebral palsy was identified in 79.9% of cases. Causes prior to the perinatal period were, compared to perinatal brain damage, associated significantly with different comorbidities. The term *cerebral palsy* does not describe a single disease but is an umbrella term covering many different diseases. Depending on the cause, a varying clinical phenotype can be found, which offers great potential in terms of individual treatment and preventing comorbidities.

## Introduction

Cerebral palsy is the most common motor disability in childhood, with a prevalence of about 2:1000 live-births and up to about 112:1000 in preterm births born before 28 weeks of gestation.^1^ The term *cerebral palsy* has been loosely defined as “a group of permanent disorders of the development of movement and posture, causing activity limitation, that are attributed to nonprogressive disturbances that occurred in the developing fetal or infant brain.”^2^ Disease outcome depends on early diagnosis and therapy,^3-5^ and the quality of life of children with cerebral palsy is strongly associated with the presence of comorbidities.^6,7^

Although first described using the term *cerebral paralysis* in 1843 by the English orthopedic surgeon William Little,^8^ there is still a huge disunity in defining cerebral palsy. Since the first description, the definition and classification of cerebral palsy has been discussed extensively, with the consensus that cerebral palsy is an umbrella term that includes a variety of clinical and etiologic aspects.^2^ Nevertheless, several surveillance programs use more than 1 cerebral palsy definition,^9^ and there is discord concerning inclusion and exclusion criteria.^10^ In addition, the surge of next-generation techniques has led to the identification of genetic causes in individuals with the official diagnosis “cerebral palsy.” This underlines the fact that 165 years after its first description there is still an uncertainty when it comes to defining cerebral palsy. Therefore, our aim was to determine to what extent the clinical phenotype of cerebral palsy patients depends on the underlying cause and discuss if *cerebral palsy* is still the proper umbrella term to cover them.

## Materials and Methods

Study type and study group: For this retrospective study, we evaluated the medical records of 384 children with cerebral palsy. These children were treated at the Center for Chronically Sick Children at Charité University Medicine Berlin, Germany, between June 2015 and June 2017. Our cohort is representative of the Berlin metropolitan area.

### Data Collection and Definitions

We used a standardized data sheet to collect data on demographic background (origin of the parents, consanguinity and affected relatives), social background of the family, pregnancy, birth, and birth complications (asphyxia, neonatal seizures, neonatal sepsis, resuscitation, etc). Information on possible relevant abnormalities during pregnancy, birth, and the neonatal period were also retrospectively collected (alcohol, nicotine, and drug consumption during pregnancy, twin-to-twin transfusion, hypoglycemia, premature birth, multiple pregnancy, intrauterine growth restriction, [pre]eclampsia, and in vitro fertilization). Furthermore, we collected information on comorbidities and results of intellectual tests, the age at cerebral palsy diagnosis, and the cerebral palsy type (unilateral spastic, bilateral spastic, ataxic and dyskinetic). The diagnosis of cerebral palsy was made by the attending neuropediatric physician. Possible causes of cerebral palsy were defined as an event that can lead to brain damage and/or proof of brain damage in addition to matching motor disabilities. Data on cMRI was available in 308 cases, genetic testing (array-CGH or chromosomal analysis) were conspicuous in 9 cases. Abnormal genetic findings associated with another known disease not associated with movement disorder (eg Klinefelter syndrome) have been excluded. Possible causes were chromosomal aberrations, brain malformations, periventricular leukomalacia, intracerebral hemorrhage, hydrocephalus, hypoxic-ischemic encephalopathy, neonatal stroke, infections, and kernicterus. Causes were not mutually exclusive. In case of patients with several causes, every cause was included into data. Patients with cerebral palsy disability but without a clear incident and/or proof of brain damage such as a pathologic cMRI were defined as cerebral palsy of unknown origin.

Patients with brain damage occurring more than 28 days following birth were excluded from this study. Patients from whom we could not find all the necessary information in the medical records were also excluded from the study (n = 6).

To categorize the level of cerebral palsy, we used the Gross Motor Function Classification System (GMFCS), which classifies the motor impairments of the lower limb in 5 levels. It spans from GMFCS 1 (patients can walk and run with limitations in balance and speed) to GMFCS 5 (an independent mobility is not possible) and is used with adaptations for age.^11^

### Statistical Analysis

All the results below refer to the whole cohort of 384 patients. The collected data were analyzed using IBM-SPSS statistics (version 24). We used the chi-square test and the Fisher exact test for categorical variables. Other variables were evaluated by using the Mann-Whitney *U* test. *P* values <.05 were considered statistically significant. We note that owing to the large number of tests based on the same data set, there is the risk that a few of the found associations, especially those with the smaller significance (*P* > .01), occur just by chance. The study was approved by the local ethics committee (no. EA2/091/16) and data security commission (AZ379/16).

## Results

**Figure 1.**
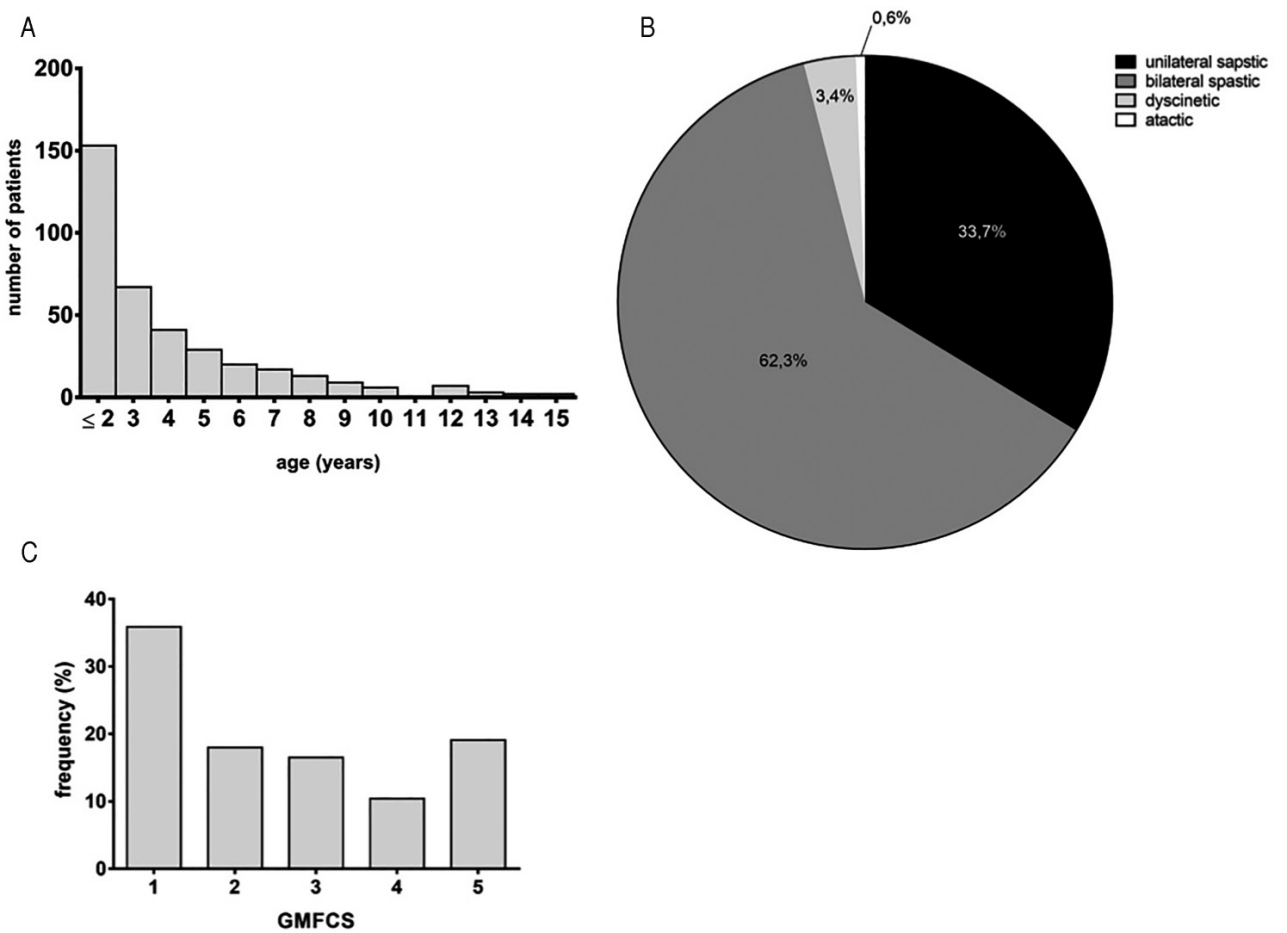
Cohort overview. (A) Age at time of cerebral palsy diagnosis (n = 384). (B) Distribution of cerebral palsy type in percentage (n = 384). (C) Distribution of GMFCS in percentage (n = 384). Abbreviations: CP ,  cerebral palsy; GMFCS,  Gross Motor Function Classification Scale.

The study cohort comprised 384 patients with a mean age of 10.62 years (SD 5.0, range 10 months–33 years) (Figure 1A) and a predominance of the male sex (63.3% male, n = 189; 36.7% female, n = 102). The median age at the time of cerebral palsy diagnosis was 3.76 years (SD 2.95, range 0-15 years). Most patients had bilateral spastic cerebral palsy (62.3%, n = 220), followed by unilateral spastic cerebral palsy (33.7%, n = 119). The dyskinetic (3.4%, n = 12) and ataxic cerebral palsy (0.6%, n = 2) subtypes were rare (Figure 1B). About a third of the patients were classified as GMFCS 1 (35.8%, n = 124); 18.2% were classified as GMFCS 2 (n = 63), 16.5% as GMFCS 3 (n = 57), 10.4% as GMFCS 4 (n = 36), and 19.1% as GMFCS 5 (n = 66) (Figure 1C).

Visual impairment was the most common comorbidity in patients with cerebral palsy (49%, n = 188). Other comorbidities identified were epilepsy (29.9%, n = 115), scoliosis (21.4%, n = 82), hip dislocation (14.6%, n = 56), swallowing disorder (14.3%, n = 55), hip dysplasia (13.3%, n = 51), hearing impairment (8.9%, n = 34), osteoporosis (1.8%, n = 7) and pathological fractures (1.6%, n = 6).

## Cerebral Palsy Causes

We identified a possible cause for cerebral palsy in 77.1% (n = 296) of the cases. In 13% (n = 50), we could not identify a specific cause, but abnormalities were reported during conception, pregnancy, and/or delivery. This leaves only 9.9% (n = 38) with a negative medical history (uneventful conception, pregnancy, and delivery).

**Figure 2.**
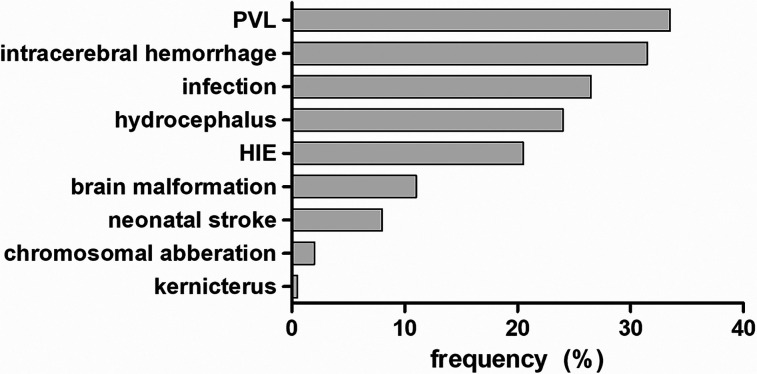
***Causes of CP.*** Distribution of cerebral palsy causes in percentage. Abbreviations: CP,  cerebral palsy; HIE , hypoxic ischemic encephalopathy; PVL,  periventricular leukomalacia.

The most common cause of cerebral palsy was periventricular leukomalacia (33.6%, n = 129) in children born prematurely, followed by intracerebral hemorrhage (32%, n = 123) and hydrocephalus (24.2%, n = 93). Other causes were hypoxic-ischemic encephalopathy (20.3%, n = 78), infection (23.9%, n = 92), brain malformations (11.2%, n = 43), neonatal stroke (7.6%, n = 29), chromosomal aberrations (2.3%, n = 9), and 1 case of kernicterus (.3%) (Figure 2). Chromosomal aberrations included microdeletions 17p13.3, 20p13, 1p36, and 3p22.1 as well as microduplications 1q32.1.

## Abnormalities During Conception, Pregnancy, and/or Delivery

We detected various abnormalities during conception, pregnancy, and/or delivery in patients with cerebral palsy (Table 1). The majority of patients were born preterm (53.9%, n = 207), and 17.7% were multiple births (n = 68). In 43 cases (11.2%), the cardiotocography result had been pathologic before or during delivery. Moreover, 9.6% (n = 37) of the children's parents were consanguine, a coagulation disorder was identified in 6% (n = 23) of the cases, 6% had been hypoglycemic, and in 4.7% (n = 18) the pregnancy was achieved through in vitro fertilization / intracytoplasmic sperm injection. In 3.9% of the cases (n = 15) the mother had a hypertensive disease of pregnancy, and 3.4% (n = 13) of the cases had a history of umbilical cord entanglement. Other abnormalities were use of nicotine during pregnancy (2.9%, n = 11), twin-to-twin-transfusion (2.6%, n = 10), cardiac arrest (2.6%, n = 10), acidosis (2.1%, n = 8), intrauterine growth restriction (1.6%, n = 6), drug use during pregnancy (1.0%, n = 4), transposition of great arteries (.5%, n = 2).

**Table 1.** Abnormalities During Conception, Pregnancy, and/or Delivery.

	n	% of cases
Premature birth	20	53.9
Part of multiple birth	68	17.7
Pathologic CTG	43	11.2
Consanguinity	37	9.6
Coagulation disorder	23	6.0
Hypoglycemia	23	6.0
IVF/ICSI	18	4.7
HDP	15	3.9
Umbilical cord entanglement	13	3.4
Nicotin	11	2.9
TTTS	10	2.6
Cardiac arrest	10	2.6
Acidosis	8	2.1
IUGR	6	1.6
Drugs	4	1.0
TGA	2	0.5

Abbreviations: CTG, cardiotocography; HDP,  hypertensive disease of pregnancy; ICSI,  intracytoplasmic sperm injection; IUGR,  intrauterine growth restriction; IVF,  in vitro fertilization; TGA,  transposition of great arteries; TTTS,  twin-to-twin transfusion.

## Associations Between Cause and Clinical Phenotype

We further correlated the clinical phenotype, abnormalities during pregnancy, and delivery with the cause of cerebral palsy (Table 2 / Supplemental Table 1). We found that children with cerebral palsy due to a genetic cause had higher GMFCS levels (*P* = .004) and more often presented with epilepsy (*P* = .04). Patients with cerebral palsy due to brain malformation had higher GMFCS levels significantly more often (*P* < .001), a dyskinetic cerebral palsy subtype (*P* = .03), lower birth weight percentiles (*P* = .02), epilepsy (*P* = .001), swallowing disorders (*P* = .002), hearing impairment (*P* = .007), hip dislocation (*P* = .009), scoliosis (*P* < .001), osteoporosis (*P* = .004), and pathological fractures (*P* = .02). In this subgroup of patients, unilateral spastic cerebral palsy (*P* = .01) and premature birth (*P* = .003) occurred less frequently. In children with hypoxic-ischemic encephalopathy, we noted a higher GMFCS (*P* < .001), a larger proportion of children with dyskinetic cerebral palsy (*P* = .01), pathologic cardiotocography (*P* = .003), cardiac arrest (*P* = .006), emergency cesarean section (*P* < .001), fetal-to-neonatal maladaptation (*P* < .001), epilepsy (*P* = .03), swallowing disorders (*P* < .001), hip dislocation (*P* = .04), and hip dysplasia (*P* = .01). Rarer on the other hand were unilateral spastic cerebral palsy (*P* = .02) and spontaneous birth (*P* = .01). In patients with periventricular leukomalacia, cerebral palsy was diagnosed significantly earlier (*P* = .001) and the maternal age at delivery was higher (*P* = .02). Additionally, periventricular leukomalacia correlated with a higher number of cases with maternal smoking during pregnancy (*P* = .04), premature birth (*P* = .001), and hip dislocation (*P* = .02). Neonatal stroke was associated with early cerebral palsy diagnosis (*P* = .02), unilateral spastic cerebral palsy (*P* < .001), spontaneous birth (*P* = .02), and vacuum extraction (*P* = .001), whereas fewer cases were found with a higher GMFCS (*P* = .03), bilateral spastic cerebral palsy (*P* < .001), multiple birth (*P* = .009), premature birth (*P* < .001), primary cesarean section (*P* = .01), fetal-to-neonatal maladaptation (*P* < .001), visual defects (*P* = .01), and scoliosis (*P* = .004). In patients with intracerebral hemorrhage, unilateral spastic cerebral palsy (*P* < .001), multiple birth (*P* = .008), premature birth (*P* < .001), fetal-to-neonatal maladaptation (*P* = .001), and coagulation disorders (*P* = .002) were significantly more frequent. On the other hand, they less often had a high GMFCS (*P* = .03), bilateral spastic cerebral palsy (*P* = .006), consanguineous parents (*P* = .03), and vacuum extractions (*P* = .02). Children with hydrocephalus were more often part of multiple birth (*P* = .003), born prematurely (*P* < .001), had fetal-to-neonatal maladaptation (*P* < .001), epilepsy (*P* = .03), swallowing disorders (*P* = .02), visual defects (*P* < .001), and hip dislocations (*P* = .03). In the group of children with infections, bilateral spastic cerebral palsy (*P* < .01), premature birth (*P* < .001), fetal-to-neonatal maladaptation (*P* < .001), coagulation disorders (*P* = .001), acidosis (*P* = .02), and visual defects (*P* = .001) occurred more often.

**Table 2.** Clinical Phenotype of Patients With Cerebral Palsy Depends on the Cerebral Palsy Cause.

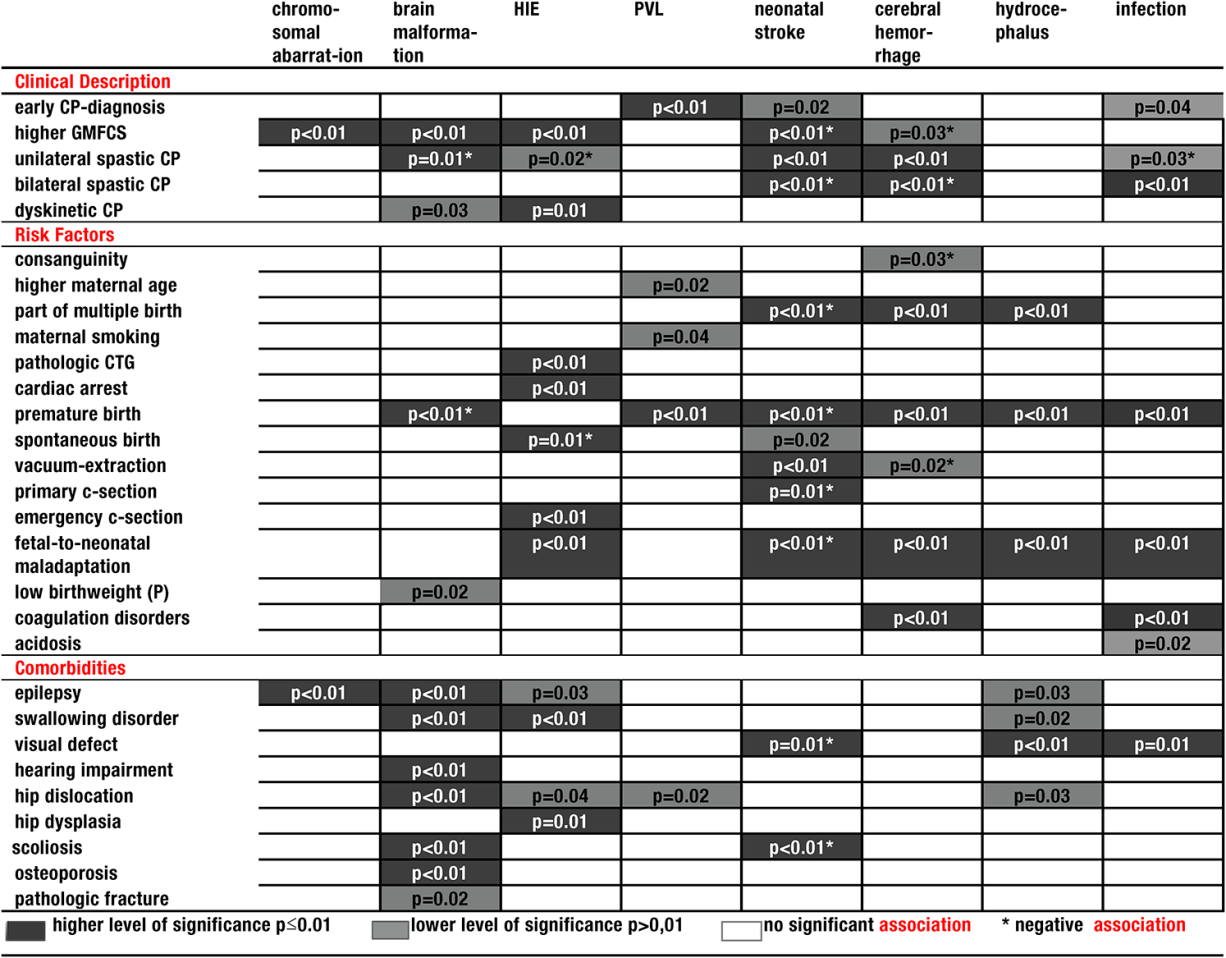								

Abbreviations: CP,  cerebral palsy; c-section,  cesarean section; CTG,  cardiotocography; GMFCS,  Gross Motor Function Classification Scale; HIE,  hypoxic ischemic encephalopathy; PVL,  periventricular leukomalacia; P,  centiles (chi-square test, Fisher exact test, Mann-Whitney *U* test).

## Discussion

The aim of this retrospective study was to identify the differences in clinical phenotype, in children with cerebral palsy depending on the etiology. Our results demonstrate that the cerebral palsy cause is a major determinant for the clinical phenotype in terms of perinatal factors, comorbidities, cerebral palsy types, and severity.

Looking at the differences in detail, one can see that a higher GMFCS is more common in children with chromosomal aberration, brain malformation, and hypoxic-ischemic encephalopathy and less common in children with neonatal stroke and cerebral hemorrhage. For children with brain malformation, this has already been described by Jystad et al.^12^

The results are also very explicit in terms of comorbidities. They clearly show that children with cerebral palsy due to chromosomal aberration, brain malformation, hypoxic-ischemic encephalopathy, and hydrocephalus are more likely to develop epilepsy. An accumulation of swallowing disorders was found in children with hypoxic-ischemic encephalopathy, brain malformations, and hydrocephalus. Visual defects, however, were found more frequently in children with hydrocephalus, and infections. Finally, hearing impairment is more common in children with brain malformations.

### Early Treatment of Motor Impairment

The question that occurs is, Should all these different “versions” of cerebral palsy still be labeled with the same term although each of them shows an individual clinical phenotype? The main argument against the term cerebral palsy comes with the possibility of improved medical support and individualized therapy depending on cause and known associated problems. Chromosomal aberration and brain malformation affect a larger part of the brain, which explains the higher GMFCS there. The main damage in patients with hypoxic-ischemic encephalopathy is located in the deep gray matter, especially in the thalamic and basal ganglia region.^13^ Although this is a circumscribed region, lesions in this part of the brain as a central coordination point of motor function can have a huge impact on motor function like extrapyramidal and dyskinetic disabilities and therefore can affect the GMFCS.^14^ On the other hand, there are neonatal strokes and intracerebral hemorrhage, which mostly affect one specific part of the brain and often occur unilaterally. This may explain why they are associated with a lower GMFCS. Thus, the cause of the cerebral palsy leads to the characteristics and severity of the motor disabilities. The treatment regarding motor abilities is diverse, including physiotherapy and occupational therapy, orthosis, walking aids or wheelchairs, surgical intervention,^15^ hippotherapy,^16^^,^^17^ power training,^18^ and botulinum toxin sessions.^19^ Treatment influences the motoric outcome of the children positively.^20^ The knowledge of the fact that the GMFCS and therefore the motor abilities of a child are linked to the cerebral palsy cause provides the opportunity to identify children who would benefit most and to initiate individual therapy at an early stage. Especially, children with milder cerebral palsy seem to benefit from early motor training.^6^

### Early Treatment of Comorbidities

Comorbidities of cerebral palsy patients in general are strongly associated with their quality of life^217^ and they therefore play a central role in the medical care of the patients. Novak et al^5^ state that screening for disabilities in orthopedics, neurologic fields, urinary tract, sleep, aural care, ophthalmologic issues, feeding issues, and aural fields could prevent secondary impairments and optimize outcomes. For example, if we know the cerebral palsy causes where epilepsy occurs more frequently, affected children could be monitored more accurately on symptoms associated with epilepsy. The accumulation of swallowing disorders in children with hypoxic-ischemic encephalopathy, brain malformations, and hydrocephalus reveals the possibility of early treatment as well. Early intervention can improve outcomes and reduce complications of swallowing disorders, as Asgarshirazi et al state in their article from 2017.^22^ Visual defects should be monitored in cerebral palsy patients with hydrocephalus and infections. Untreated hearing impairment can lead to severe impairment in communication^23^ and should therefore be treated as early as possible. Morgan et al therefore suggest in their study from 2017 that it would be necessary to manage comorbidities in order to optimize outcomes and prevent secondary impairments.^6^ Therefore, focusing on the cause of cerebral palsy instead of focusing on the cerebral palsy diagnosis could provide an improved medical care for the individual patient.

### Cerebral Palsy as a Vague Definition

Another argument against the cerebral palsy diagnosis is the still vague definition of the disease. Since the first description of the term *cerebral paralysis* by William Little in 1843,^8^ the definition of cerebral palsy has often been discussed. Today, the inclusion and exclusion criteria are still not clearly defined.^10^ For instance, there is no consensus on whether or not to include neurologic syndromes that have spastics as a symptom^24^ or on an upper age limit for post-neonatal cerebral palsy.^2^ The uncertainty regarding the definition of the cerebral palsy diagnosis often leads to delayed diagnosis, which itself can lead to delayed intervention.^6^ In the past, early diagnosis of cerebral palsy was not recommended. Cans et al^25^ even defined the age of 5 years as the optimal age to confirm diagnosis. Recently, there has been a rise in the demand for early diagnosis of cerebral palsy, and diagnostic schemes have been developed to ensure that. Nevertheless, magnetic resonance imaging (MRI; as a tool to discover the cause) is still of great importance in combination with clinical motor assessments like, for example, Hammersmith Infant Neurological Examination (HINE) and Prechtl Qualitative Assessment of General Movements (GMs).^5^ This can be an important development toward early treatment, although these assessments focus primarily on motor impairment. Screening for comorbidities is only recommended later on,^5^ which makes a multidisciplinary approach difficult.

In 1998 already, Badawi et al had recognized cerebral palsy as an outdated term in consideration of the growing knowledge and improved diagnostic technologies.^24^ Smithers-Sheedy et al^10^ state in their article from 2014 that the term *cerebral palsy* resulted from the limited knowledge of etiology and pathology. With years of research, our knowledge on cerebral palsy has expanded greatly. In our cohort, we identified a cause for cerebral palsy in the majority of cases, and every cerebral palsy cause presented itself with a different clinical phenotype. Although the term *cerebral palsy* is recognized and established in pediatric neurology, as Badawi et al give as an argument in favor of the term *cerebral palsy*,^24^ this is not reason enough to hold on to an outdated diagnosis, especially if its reconsideration could improve the outcome of cerebral palsy. We state that using the causes as the diagnosis and describe paralysis, epilepsy, hearing disorders etc as a symptom or complication of the disease would be more accurate than using the umbrella term *cerebral palsy*. Detecting pathologic pathways combined with a thorough clinical assessment could improve individual treatment and intervention and thus the outcome of the patient. This would be much more accurate, and additionally avoid the uncertainty about the diagnosis of cerebral palsy and therefore prevent delayed diagnosis and treatment. Nevertheless, we are aware that *cerebral palsy* is an internationally used term that will continue to be used in international medicine that simplifies communication between health care providers. The awareness among members of the health care system should be raised that cerebral palsy is much more complex than it may seem.

### Limitations

Owing to the retrospective study design, it was not possible to demonstrate pathologic pathways or prove causation. This should be the goal of additional prospective studies, which would be an important and interesting addition to our findings. Additionally, further studies with larger cohorts must be carried out to confirm our findings.

## Conclusion

Cerebral palsy cause is a major determinant for the clinical phenotype regarding perinatal factors, comorbidities, cerebral palsy types, and severity. Our findings indicate the importance of treating children with motor disability more individually, depending on the underlying cause of their condition and their clinical phenotype.

## Supplemental Material

sj-doc-1-jcn-10.1177_08830738211059686 - Supplemental material for Clinical Phenotype of Cerebral Palsy Depends on the Cause: Is It Really Cerebral Palsy? A Retrospective Study Click here for additional data file. Supplemental material, sj-doc-1-jcn-10.1177_08830738211059686 for Clinical Phenotype of Cerebral Palsy Depends on the Cause: Is It Really Cerebral Palsy? A Retrospective Study by Charlotte Metz, Monika Jaster, Elisabeth Walch, Akosua Sarpong-Bengelsdorf, Angela M. Kaindl and Joanna Schneider in Journal of Child Neurology
