# Supplementary material for: Clinical Phenotype of Cerebral Palsy Depends on the Cause: Is It Really Cerebral Palsy? A Retrospective Study
Source: J Child Neurol. 2021 Dec 13;37(2):112–8. doi: 10.1177/08830738211059686 (PMC8804944; doi:10.1177/08830738211059686)
Supplement: sj-doc-1-jcn-10.1177_08830738211059686 - Supplemental material for Clinical Phenotype of Cerebral Palsy Depends on the Cause: Is It Really Cerebral Palsy? A Retrospective Study [file sj-doc-1-jcn-10.1177_08830738211059686.doc]

*Supplemental Table 1 Clinical phenotype of patients with CP in relation to CP cause.* Negative associations are marked by*, all other associations are positive associations. Percentages refer to the total number of the specific cause. Abbreviations: CP = cerebral palsy, HIE = hypoxic ischemic encephalopathy, PVL = periventricular leukomalacia, CTG = cardiotocography, c-section = cesarean section, P = centiles (Chi-Square test, Fisher’s exact test)

|  | **chromosomal abarration (n=9)** | **brain malformation (n=43)** | **HIE (n=78)** | **PVL (n=129)** | **neonatal stroke (n=29)** | **cerebral hemorrhage (n=123)** | **hydro-cephalus (n=93)** | **infection (n=92)** |
| --- | --- | --- | --- | --- | --- | --- | --- | --- |
| **Clinical Description** | | | | | | | | |
| **unilateral spastic CP** |  | p=0.01* n=37 86,0% | p=0.02* n=62 69,5% |  | p<0.01 n=23 79,3% | p<0.01 n=53 43,1% |  | p=0.03 * n=71 77,2% |
| **bilateral spastic CP** |  |  |  |  | p<0.01* n=25 86,2% | p<0.01* n=65 52,8% |  | p<0,01 n=63 68,5% |
| **dyskinetic CP** |  | p=0.03 n=4 9,3% | p=0.01 n=6 7,7% |  |  |  |  |  |
| **Risk Factors** | | | | | | | | |
| **consanguinity** |  |  |  |  |  | p=0.03* n=117 95,1% |  |  |
| **part of multiple birth** | ? |  |  |  | p<0.01* n=29 100% | p<0.01 n=31 25,2% | p<0.01 n=26 28,0% |  |
| **maternal smoking** |  |  |  | p=0.04 n=7 5,4% |  |  |  |  |
| **pathologic CTG** |  |  | p<0.01 n=16 20,5% |  |  |  |  |  |
| **cardiac arrest** |  |  | p<0.01 n=6 7,7% |  |  |  |  |  |
| **premature birth** |  | p<0.01* n=29 67,4% |  | p<0.01 n=85 65,9% | p<0.01* n=25 86,2% | p<0.01 n=93 75,6% | p<0.01 n=67 72,0% | p<0.01 n=73 79,3% |
| **spontaneous birth** |  |  | p=0.01* n=59 75,6% |  | p=0.02 n=16 55,2% |  |  |  |
| **vacuum-extraction** |  |  |  |  | p<0.01 n=6 20,7% | p=0.02* n=122 99,2% |  |  |
| **primary c-section** |  |  |  |  | p=0.01* n=28 96,6% |  |  |  |
| **emergency c-section** |  |  | p<0.01 n=21 27,0% |  |  |  |  |  |
| **fetal-to-neonatal maladaptation** |  |  | p<0.01 n=21 65,4% |  | p<0.01* n=26 89,7% | p<0.01 n=67 54,55 | p<0.01 n=52 55,9% | p<0.01 n=61 66,3% |
| **coagulation disorders** |  |  |  |  |  | p<0.01 n=14 11,4% |  | p<0.01 n=11 12,0% |
| **acidosis** |  |  |  |  |  |  |  | p=0.02 n=5 5,4% |
| **Comorbidities** | | | | | | | | |
| **epilepsy** | p<0.0 1 n=77 77,8% | p<0.01 n=22 51,2% | p=0.03 n=31 39,7% |  |  |  | p=0.03 n=36 38,7% |  |
| **swallowing disorder** |  | p<0.01 n=13 30,2% | p<0.01 n=21 26,9% |  |  |  | p=0.02 n=20 21,5% |  |
| **visual defect** |  |  |  |  | p=0.01* n=21 72,4% |  | p<0.01 n=65 69,9% | p=0.01 n=62 67,4% |
| **hearing impairment** |  | p<0.01 n=9 20,9% |  |  |  |  |  |  |
| **hip dislocation** |  | p<0.01 n=12 28,0% | p=0.04 n=17 21,8% | p=0.02 n=26 20,2% |  |  | p=0.03 n=20 21,5% |  |
| **hip dysplasia** |  |  | p=0.01 n=17 21,8% |  |  |  |  |  |
| **scoliosis** |  | p<0.01 n=19 44,2% |  |  | p<0.01* n=29 100% |  |  |  |
| **osteoporosis** |  | p<0.01 n=4 9,3% |  |  |  |  |  |  |
| **pathologic fracture** |  | p=0.02 n=3 7,0% |  |  |  |  |  |  |
| |  |  |  |  |  |  | | --- | --- | --- | --- | --- | --- | |  | 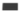 | higher level of significance p≤0.01 | 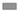 | lower level of significance p>0,1 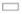 no significant association * negative association |  | |  |  |  |  |  | | | | | | | | | |
